# Supplementary material for: Y-box protein 1 is required to sort microRNAs into exosomes in cells and in a cell-free reaction
Source: eLife. 2016 Aug 25;5:e19276. doi: 10.7554/eLife.19276 (PMC5047747; doi:10.7554/eLife.19276)
Supplement: Figure 2—source data 1. — Reads were processed (see Materials and methods) and mapped to the human genome (hg19) using Bowtie 2. Total counts for reads mapped to the genome, to rRNA and to miRNA (using miRdeep2 - see Materials and methods) are shown. Percent of total reads are shown in parenthesis. DOI: http://dx.doi.org/10.7554/eLife.19276.006 [file elife-19276-fig2-data1.docx]

| Source | Mapped Reads | rRNA | miRNA | | |
| --- | --- | --- | --- | --- | --- |
| 293T Cells | 12089110  **(100%)** | 7905728  **(65.4%)** | 880093 | | |
|  |  |  | w/rRNA  (7.3%) | w/o rRNA  (21.0%) | |
| 293T Exosomes | 2811327  **(100%)** | 2242164  **(79.8%)** | 123679 | | |
|  |  |  | w/ rRNA  (4.4%) | | w/o rRNA  (21.7%) |

**Figure 2 - source data 1: Mapping statistics for small RNA-seq libraries to the human genome (hg19)**
